# Which Neurodevelopmental Disorders Get Researched and Why?

**DOI:** 10.1371/journal.pone.0015112

**Published:** 2010-11-30

**Authors:** Dorothy V. M. Bishop

**Affiliations:** Developmental Neuropsychology, University of Oxford, Oxford, United Kingdom; University of Giessen Lung Center, Germany

## Abstract

**Aim:**

There are substantial differences in the amount of research concerned with different disorders. This paper considers why.

**Methods:**

Bibliographic searches were conducted to identify publications (1985–2009) concerned with 35 neurodevelopmental disorders: Developmental dyslexia, Developmental dyscalculia, Developmental coordination disorder, Speech sound disorder, Specific language impairment, Attention deficit hyperactivity disorder, Autistic spectrum disorder, Tourette syndrome, Intellectual disability, Angelman syndrome, Cerebral palsy, Cornelia de Lange syndrome, Cri du chat syndrome, Down syndrome, Duchenne muscular dystrophy, Fetal alcohol syndrome, Fragile X syndrome, Galactosaemia, Klinefelter syndrome, Lesch-Nyhan syndrome, Lowe syndrome, Marfan syndrome, Neurofibromatosis type 1, Noonan syndrome, Phenylketonuria, Prader-Willi syndrome, Rett syndrome, Rubinstein-Taybi syndrome, Trisomy 18, Tuberous sclerosis, Turner syndrome, Velocardiofacial syndrome, Williams syndrome, XXX and XYY. A publication index reflecting N publications relative to prevalence was derived.

**Results:**

The publication index was higher for rare than common conditions. However, this was partly explained by the tendency for rare disorders to be more severe.

**Interpretation:**

Although research activity is predictable from severity and prevalence, there are exceptions. Low rates of research, and relatively low levels of NIH funding, characterise conditions that are the domain of a single discipline with limited research resources. Growth in research is not explained by severity, and was exceptionally steep for autism and ADHD.

## Introduction

It is well recognised that there are substantial differences in the amount of research activity concerned with different medical conditions, but the reasons for disparities are not fully understood. Within the field of neurology, Al-Shahi and colleagues found that the amount of research into a condition was not proportionate to the frequency of the disease [1]. Common conditions such as stroke or migraine were under-researched relative to rare diseases such as Wilson's disease or Creutzfeldt-Jakob disease, when assessed using ‘publication ratio’, a measure of number of publications divided by disease frequency. They suggested that as well as political, economic and scientific interest, research activity might also be influenced by the fashionable nature of some conditions, and recommended that similar analyses should be conducted in other areas. However, their analysis did not take into account the severity of disease, which might be expected to play some part in research priorities. A review of grants awarded by the National Institutes of Health [2] confirmed that research funding was not related to how common a disease was, but it was related to disease burden, as indicated by mortality, years of life lost, and disability-adjusted life years. Thus severity of the impact of a condition seemed to play a larger role in determining research priorities than disease frequency.

The field of neurodevelopmental disorders is a microcosm in which these issues can be further explored. The term neurodevelopmental disorder is used in two ways [3]. The first refers to conditions affecting children's neurological development with a known genetic or acquired etiology, such as Fragile X syndrome or fetal alcohol syndrome. The second use refers to conditions of presumed multifactorial etiology in which certain aspects of neurodevelopment are selectively impaired; this includes such conditions as autistic disorder, developmental dyslexia, and attention-deficit hyperactivity disorder (ADHD). Both types of condition were included in the current analysis. In this article, counts are provided for publications in this field to consider how far the amount of a research on a condition can be explained by (a) frequency of disorder, (b) severity of disorder or (c) the disciplines involved in research. In addition, research activity is evaluated in terms of funding by the National Institutes of Health (NIH) relating to these conditions.

## Methods

### Conditions included in the analysis

The conditions included in this review are shown in Table 1. These were identified from Rutter's Textbook of Child and Adolescent Psychiatry, 5th edition [4] and a review of behavioural phenotypes [5]. Those from the latter source were included only if an estimate of prevalence was available. The category of ‘intellectual disability’ (ID) posed problems, because this can be both a symptom of a known disorder, and a nonsyndromal condition of unknown etiology. Furthermore, in the UK, the term ‘learning disability’ is used to refer to intellectual disability, whereas elsewhere ‘learning disability’ is used for specific difficulties in a child of normal IQ. Despite these problems, it was decided to attempt an estimate of publications focusing on ID, but data on this condition need to be treated with particular caution.

**Table 1:** Search terms (in title) used to identify neurodevelopmental disorders.

*Presumed multifactorial etiology*	*Known etiology*
“Developmental dyslex*”	**“Angelman syndrome”**
OR dyslex* NOT (acquired OR deep OR surface)	“Cerebal pals*”
OR “specific reading disab*/retard*/impair*/difficulties”	**“Cornelia de Lange syndrome”**
OR “developmental reading disorder*”	OR “Brachmann-de Lange”
“Developmental dyscalcul*”	**“Cri du chat syndrome”**
OR “specific arithmetic*/math* disab*/retard*/disorder*”	OR “deletion 5p”
NOT dyscalcul*	**Down syndrome1**
“Developmental coordination disorder**”	“trisomy 21”
OR “developmental co-ordination disorder*”	**“Duchenne muscular dystrophy”**
OR "developmental dysprax*	**“Fetal alcohol syndrome”**
OR “clumsy child*” NOT dysprax*	“foetal alcohol syndrome”
“Speech sound disorder*”	**“Fragile X syndrome” OR FraX**
OR “articulation difficulties” AND child* NOT palate	**Galactosemia OR Galactosaemia**
OR “articulation disorder*” AND child*	“Klinefelter* syndrome” OR XXY
OR “apraxia of speech” AND (childhood OR developmental)	**“Lesch-Nyhan syndrome”**
OR (“delayed speech” OR “speech delay”) NOT palate	“Lesch Nyhan syndrome”
OR “developmental apraxia of speech”	**“Lowe/Lowe's syndrome”**
OR “developmental articulation/phonological disab*/disorder*”	**“Marfan syndrome”**
OR “developmental phonological impair*/problem*”	**“Neurofibromatosis type ** 1”
OR “developmental verbal dyspraxia”	“Neurofibromatosis type I”
OR “phonological disabi*/disorder*/impair*problems” AND child* NOT developmental	**“Noonan syndrome”**
OR “speech difficulties/disorder*/impair*” AND child*	**Phenylketonuria OR PKU**
OR “phonological disabi*/disorder*/impair*problems” AND child* NOT developmental	**“Prader-Willi syndrome”**
OR “speech difficulties/disorder*/impair*” AND child*	**“Rett/Rett's syndrome”**
“Specific language impair*”	**“Rubinstein-Taybi syndrome”**
OR “developmental dysphas*/aphas*”	**“Smith-Magenis syndrome”**
OR “developmental language” AND	**“Trisomy 18”**
disorder*/impair*disab*/difficult*	OR “Edwards syndrome”
OR SLI NOT specific impairment potato* gene elegans battery	**“Tuberous sclerosis”**
flight arithmetic SLI-1 crystal BMT laser somatostatin gastrin	“Turner* syndrome” NOT Ullrich
OR “language learning disab*”	**“Velocardiofacial syndrome”**
“Attention deficit hyperactivity disorder*”	OR “Velo-cardio-facial syndrome”
OR “attention deficit” NOT (hyperactiv* OR schiz*)	OR “22q11.2 deletion”
OR ADHD NOT “attention deficit”	**“Williams syndrome”**
**Autism OR autistic**	OR “Williams-Beuren syndrome”
OR (Asperger OR PDDNOS OR “pervasive developmental	OR “idiopathic infantile
disorder”) NOT autis*	hypercalcaemia/calcemia”
“Tourette* syndrome”	**XXX** AND (female* OR karyotype
**“Intellectual disability/handicap/retardation”**	OR chromosome OR trisomy)
OR “mental handicap/retardation”	**XYY**
OR “learning disab*” NOT specific	

Term in bold is main category; plain text beneath indicates alternative search terms. Asterisk is wildcard symbol.

1 Estimated from rate for ‘trisomy 21’: see text.

### Number of people affected

Estimates of prevalence per 100 were computed from Rutter et al. [4] or Udwin and Dennis [5], with the mean being used if a range was given.

### Severity of condition

Measures of disease burden used in mainstream medicine focus mainly on mortality and morbidity and are not in general appropriate for neurodevelopmental disorders. It was therefore necessary to derive an ad hoc measure for this study. This was a 4-point scale representing the extent to which an affected individual could be expected to obtain educational qualifications and live independently in adulthood (see Table 2). Nine expert clinicians who saw children with neurodevelopmental disorders were asked to estimate severity for conditions shown in Table 1 on this scale. For some conditions, this scale was difficult to apply because of the wide variation in severity. For instance, a person with autistic disorder is unlikely to fall in category 1, but could fall into category 2, 3 or 4. In such a case, raters were asked to give their best estimate of the average level of severity. The data in Table 3 shows the average across all raters. For rare conditions, few clinicians had sufficient experience of cases to be able to make a judgement, and in those cases, the author provided a rating based on the description of the phenotype in Udwin and Dennis [5].

**Table 2:** Coding of severity of impact of condition.

1	Little impact on ability to obtain educational qualifications and live independently
2	Likely to have poorer than average educational qualifications but can live independently
3	Likely to require special schooling; may be employable in adulthood, but likely to need support in daily living
4	Likely to require full-time care and support during childhood and adulthood

**Table 3:** Prevalence per 100, severity rating, number of publications (1985–2009), rate of increase in publications, % publications with genetic/animal model content, estimated N affected children in the UK, and publication index for each condition, ordered by prevalence.

Condition	Prevalence per 100^1^	Mean severity	N pubs in 25 yr	Rate of increase^2^	% genet.	N cases in UK	Pub. index^3^
Lesch-Nyhan syndrome	0.0005	4.00	428	−14	69	57	750.88
Lowe syndrome	0.0005	4.00	171	1	67	57	300.00
Rubinstein-Taybi syndrome	0.0008	3.50	291	2	48	92	316.30
Cornelia de Lange syndrome	0.0014	4.00	420	20	54	165	254.55
Cri du chat syndrome	0.0020	4.00	162	1	79	231	70.13
Galactosaemia	0.0020	2.50	678	8	46	231	293.51
Angelman syndrome	0.0040	3.79	690	36	80	462	149.35
Williams syndrome	0.0044	3.31	1,349	87	54	513	262.96
Marfan syndrome	0.0067	1.50	1,805	71	53	770	234.42
Prader-Willi syndrome	0.0067	3.17	1,851	82	58	770	240.39
Rett syndrome	0.0080	3.94	1,918	35	59	924	207.58
Phenylketonuria	0.0100	2.00	2,941	9	51	1,156	254.41
Duchenne muscular dystrophy	0.0143	2.50	3,212	−28	64	1,651	194.55
Tuberous sclerosis	0.0167	2.69	3,083	76	42	1,926	160.07
Trisomy 18	0.0250	3.70	661	−1	75	2,890	22.87
Velocardiofacial syndrome	0.0250	2.72	839	83	82	2,890	29.03
Neurofibromatosis type 1	0.0308	2.00	1,840	151	62	3,556	51.74
Turner syndrome	0.0400	1.94	2,485	21	60	4,624	53.74
XYY	0.0545	2.00	251	−6	86	6,300	3.98
XXX	0.0550	1.50	62	−3	87	6,358	0.98
Noonan syndrome	0.0571	2.50	635	44	67	6,605	9.61
Fragile X syndrome	0.0615	3.57	4,008	60	81	7,113	56.35
Klinefelter syndrome	0.0860	1.83	1,312	29	64	9,941	13.20
Fetal alcohol syndrome	0.1000	2.58	1,105	14	16	11,560	9.56
Cerebral palsy	0.1500	2.50	6,988	334	1	17,340	40.30
Down syndrome	0.1667	3.44	15,522	295	70	19,266	80.57
Tourette syndrome	0.5000	1.25	2,071	24	27	57,800	3.58
Autistic spectrum disorder	0.6500	2.90	16,071	1,468	18	75,140	21.39
Developmental dyscalculia	3.0000	1.56	229	15	14	346,800	0.07
Attention deficit hyperactivity disorder	5.0000	1.95	12,631	1,434	13	578,000	2.19
Intellectual disability^4^	5.5000	2.75	17,721	497	19	635,800	2.79
Developmental dyslexia	6.0000	1.90	3,789	128	10	693,600	0.64
Developmental coordination disorder	6.5000	1.50	398	38	0	751,400	0.05
Specific language impairment	7.4000	2.15	1140	84	8	855,440	0.13
Speech sound disorder	10.0000	1.69	387	24	11	1,156,000	0.03

1 Prevalence has been divided by two if reported for one sex only.

2 Average N additional publications per 5 yr period.

3 N publications in 25 yr per 100 affected cases in UK, based on population of 11.56 million children.

4 Includes ‘learning disability’ if not specified as ‘specific’ (see text).

### Estimates of number of publications

Web of Knowledge was the basis for research counts; the search terms used are shown in Table 1; for a paper to be counted, the search term had to appear in the title. This exercise revealed that some disorders lacked consistent terminology and were correspondingly hard to search. Total number of articles for each condition was obtained for 5-year bands from 1985 to 2009. Note that coding of articles was not mutually exclusive, so that if search terms for two conditions were included in the title, the same article would be counted towards toward the total for both conditions. The word ‘Down’ is not a valid search term in Web of Knowledge, so it was necessary to estimate the number of articles on Down syndrome. From a Google scholar search it was established that 9.9% of articles on Down syndrome include ‘trisomy 21’ in the title. Thus the number of articles on Down syndrome in Web of Knowledge was estimated by multiplying the number of articles with ‘trisomy 21’ in the title by 10.1.

### Publication index

This was derived in a method similar to that used by Al-Shahi et al [1] by dividing the total number of articles by prevalence. This was scaled so that it could be readily interpreted as a count of the N papers in 25 years per 100 affected cases in a population of 11.56 million, which is an estimate of number of children in the UK [6].

### Rate of increase in publications

This measure was derived by computing the slope of the line linking the number of publications in 5-year time bins across the period 1985–2009.

### Estimates of amount of genetic/animal research

Preliminary analyses suggested that the amount of research on a condition was partly determined by the disciplines involved; for some conditions, a high proportion of studies involved genetics and/or mouse models. To gain an estimate of how much research on each condition was of this type, searches were re-run to find the proportion of papers on a condition that had the words ‘gene’, ‘genetic’, ‘mouse’, ‘mice’ or ‘chromosome’ in the topic field.

### Amount of funding by the National Institutes of Health (NIH)

Data on NIH funding were obtained using the Research Portfolio Online Reporting Tools (RePORT) (http://projectreporter.nih.gov/reporter.cfm). For each disorder, the database was interrogated to identify funded projects for the period 2000–2010, and the total funding in thousands of dollars was computed, together with the NIH institute that provided the funds. It is important to note that the RePORT software identifies projects on the basis of keywords, and may include projects that do not have a central focus on the disorder in question. This means that, on the one hand, the amount of funding focused on a specific disorder is likely to be over-estimated, and on the other hand, that the same project is likely to be included in counts for more than one disorder. For this analysis, it proved impossible to obtain realistic estimates of funding for studies of intellectual disability, as the search terms from Table 1 identified numerous projects concerned with a wide range of disorders. This category was therefore omitted from this analysis. For the remaining conditions, a measure of rate of increase in funding between 2000–2010 was computed from the slope of the function for funding by year.

## Results

Table 3 shows the estimates of prevalence, severity of condition, number of publications, rate of increase in publications, proportion of genetic/animal papers for each condition, and publication index, with disorders ranked by prevalence. Most variables were skewed, and so nonparametric Spearman correlations were used when assessing association between variables.

The first point to note is that the publications count has only a weakly significant relationship to prevalence of a condition (r_s_ = .34, N = 35, p = .046). To explore predictors of publications, the publication index was used, i.e. a measure that reflects the number of publications relative to estimated number of affected individuals. It is evident on inspection that there is a general trend for a higher publication index for the rarer conditions; the Spearman correlation between publication index and prevalence is r_s_ = −.91, N = 35, p<.001. However, when we consider the common conditions, we see that most of them have a relatively low severity index; indeed, there is a significant negative correlation between severity and prevalence, r_s_ = −.61, N = 35, p<.001. Furthermore, the severe, rare conditions are more likely than common, milder conditions to be monogenic disorders, consistent with the fact that they are likely to have a high proportion of publications including terms indicative of genetic studies; the correlation between % genetic papers and prevalence was r_s_ = −.52, N = 35, p = .001. The correlation between % genetic papers and severity was also significant: r_s_ = .41, N = 35, p = .015.

**Figure 1:**
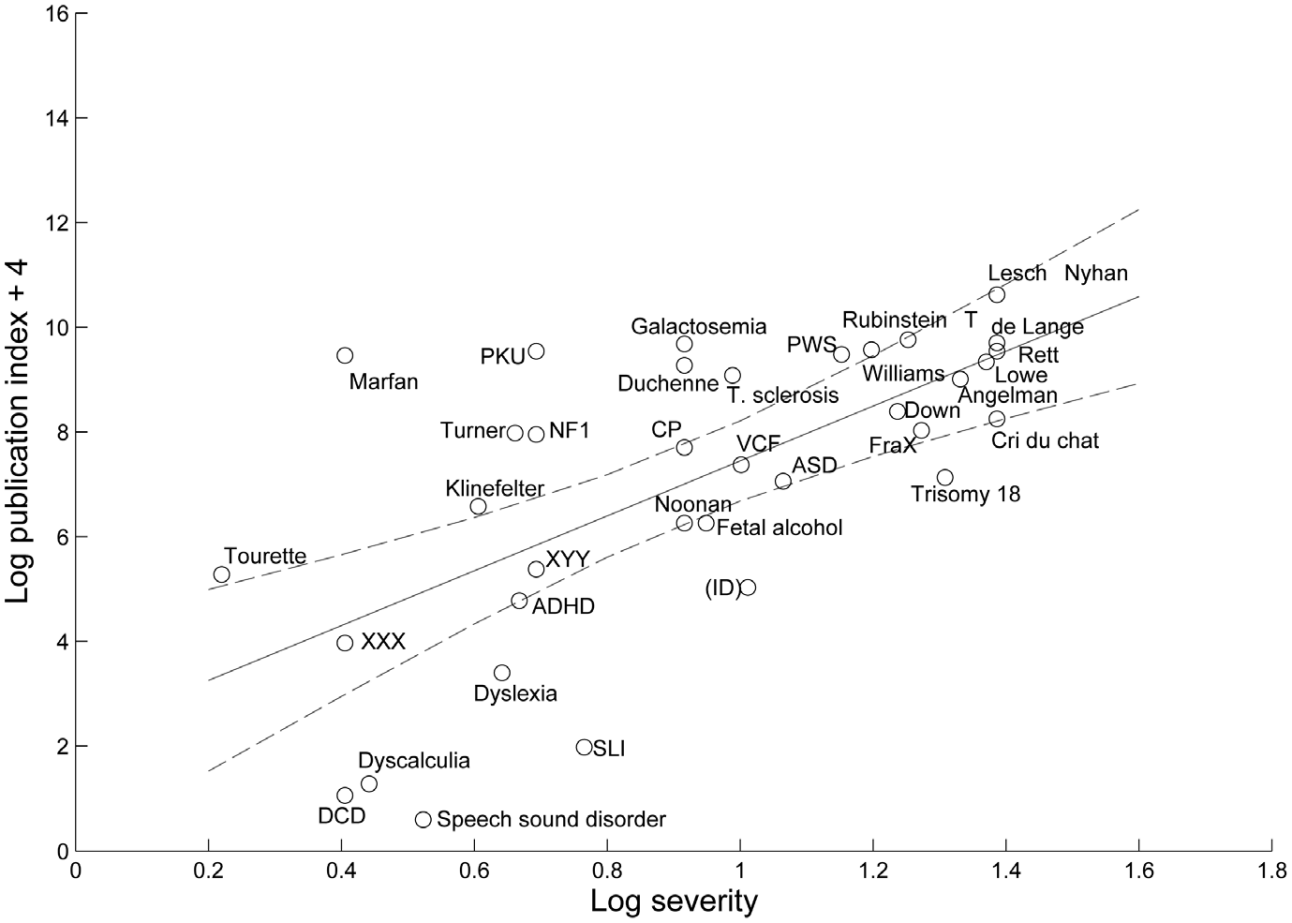
Regression of log publication index on log severity, with 95% confidence interval shown with dotted lines. A constant of 4 is added to log publication index to avoid negative numbers. Abbreviations: ADHD: attention deficit hyperactivity disorder; ASD: autism spectrum disorder; CP: cerebral palsy; DCD: developmental co-ordination disorder; de Lange: Cornelia de Lange syndrome; FraX: fragile X; ID: intellectual disability (shown in brackets to indicate that the publication index is overestimated); NF1: neurofibromatosis type 1; PKU: phenylketonuria; SLI: specific language impairment; T. sclerosis: tuberous sclerosis; VCF: velocardiofacial syndrome.

Further analysis was conducted to consider how far the publication index for each condition related to severity and genetic content. A regression analysis was conducted to find the best-fitting function relating severity to publication index, and a log-log relationship gave the best fit, with R = .648, p<.001. Figure 1 shows the scatterplot relating mean log severity to log publication index, together with the line showing predicted log publication index.

Adding the proportion of genetic papers as an independent variable led to a significant increase of fit to give R = .728; change in fit: F (1,32) = 7.51 p = .01. Adding prevalence to the model after including severity and genetic index, accounted for a further 24.3% of the variance, change in fit: F (1,31) = 33.24, p<.001, bring the multiple R up to .879.

The rate of increase in publications across the five 5-year age bands is logically independent from the publication index. This measure revealed two clear outliers, autistic spectrum disorder, and ADHD (see Table 3). Autism started from a high baseline, with 1215 publications during 1985–1989, increasing 5-fold by 2005–2009. The increase for ADHD was even more dramatic, from 356 publications in 1985–1989 to 6158 in 2005–2009. In general, rate of increase in publications was positively correlated with N publications in 1985–1989, r_s_ = .35, N = 35, p = .042, but a high initial level of publications did not guarantee rapid growth. For instance, Down syndrome started in 1985–1989 with 2121 publications, more than either ADHD or autism, but this had risen only to 3474 by 2005–2009. It is noteworthy also that Lesch-Nyhan syndrome, which had a remarkably high publication index, showed a slight decrease in number of publications over a 25 year period.

**Figure 2:**
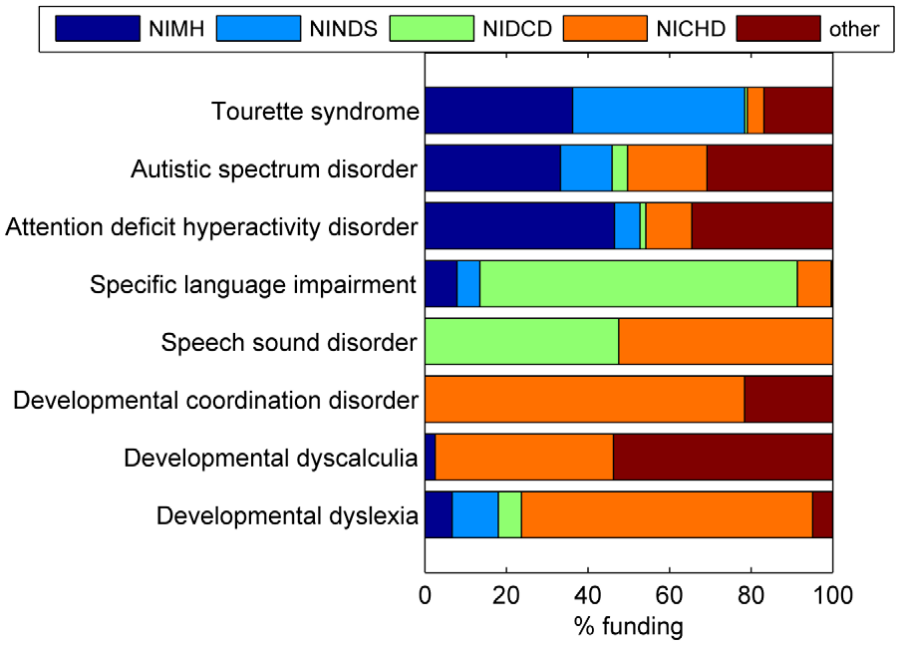
Proportion of grant income from different NIH institutes for the most common neurodevelopmental disorders. Abbreviations: NIMH: National Institute of Mental Health; NINDS: National Institute of Neurological Disorders and Stroke; NIDCD: National Institute on Deafness and Other Communication DIsorders; NICHD: Eunice Kennedy Shriver National Institute of Child Health and Human Development.

Table 4 shows data on NIH funding per condition by year, and the slope of the function. There are striking parallels with the data on publications. The Spearman correlation between the total N publications from Table 3 and the total funding from Table 4 is .883 (N = 34, p<.0001) and the correlation between the slope indicating increase in publications from 1985 to 2009, and the slope of increase in NIH funding from 2000 to 2010 is .522 (N = 34, p = .002). In a final analysis, the proportion of funding from different NIH institutes was computed for the most common neurodevelopmental disorders. These data are shown in Figure 2.

**Table 4:** Data from NIH RePort showing thousands of US dollars spent on projects relating to each disorder.

	2000–2001	2002–2003	2004–2005	2006–2007	2008–2009	Anuual rate of increase 2000–2010
Developmental dyslexia	18,770	18,199	19,971	22,975	27,283	633
Developmental dyscalculia	0	0	400	369	1,574	151
Developmental coordination disorder	0	623	602	1,379	1,166	55
Speech sound disorder	858	876	501	964	1,116	8
Specific language impairment	16,279	16,219	15,389	18,133	28,611	780
Attention deficit hyperactivity disorder	274,500	346,039	314,232	365,207	532,800	13,042
Autistic spectrum disorder	95,114	171,707	360,765	355,458	851,270	50,978
Tourette syndrome	35,604	42,119	37,031	41,626	59,587	1,150
Angelman syndrome	13,039	12,103	11,156	12,392	21,246	594
Cerebral palsy	18,197	31,412	49,467	66,507	94,578	4,409
Cornelia de Lange syndrome	194	655	677	3,541	5,789	352
Cri du chat syndrome	0	0	0	0	0	25
Down syndrome	52,067	56,561	47,662	63,890	107,390	3,924
Duchenne muscular dystrophy	7,953	14,770	33,192	47,138	62,363	3,582
Fetal alcohol syndrome	40,955	51,783	69,504	83,447	105,668	3,905
Fragile X syndrome	28,316	44,054	54,187	67,584	125,661	5,532
Galactosaemia	2,802	5,737	4,398	4,416	6,060	371
Klinefelter syndrome	686	1,049	329	4,936	9,333	513
Lesch-Nyhan syndrome	1,030	1,803	2,049	1,960	6,601	240
Lowe syndrome	2,335	2,488	3,582	2,101	1,581	−66
Marfan syndrome	3,426	4,825	5,701	9,847	20,455	872
Neurofibromatosis type 1	5,966	9,182	11,625	20,348	30,267	1,230
Noonan syndrome	0	1,138	9,099	10,615	8,650	546
Phenylketonuria	8,782	9,015	6,047	9,426	5,941	−151
Prader-Willi syndrome	14,284	15,512	17,924	17,042	24,802	620
Rett syndrome	5,194	10,235	14,980	24,650	48,575	2,995
Rubinstein-Taybi syndrome	1,023	2,766	2,304	2,648	6,032	225
Trisomy 18	0	0	578	282	0	0
Tuberous sclerosis	10,139	16,243	22,844	39,132	68,944	3,181
Turner syndrome	3,766	6,068	9,175	17,475	7,292	255
Velocardiofacial syndrome	5,042	7,229	6,850	5,632	3,895	−107
Williams syndrome	12,652	17,697	19,506	15,627	18,180	115
XXX	0	0	0	285	4,927	161
XYY	0	384	0	3,239	5,050	329

## Discussion

Before discussing the results, it is important to note that the range of conditions investigated is not comprehensive, and the approach adopted here could be extended to include a wider range of disorders. For instance, psychiatric conditions such as antisocial behavior disorder and schizophrenia were not included here, though a case can be made for treating them as neurodevelopmental disorders [3]. Reliance on a textbook of behavior phenotypes as a source book also meant that many neurological disorders without clear genetic etiology, such as congenital hypothyroidism or spina bifida, were also excluded.

It is also important to note that estimates of all of the main variables in this analysis are imprecise. A frustrating feature of this study was that is was hard to be confident that search terms identified all relevant papers without including papers on other conditions. Subsets of identified papers were scrutinised to ensure that they reflected the intended content, but relevant articles would be missed if they did not include the search terms in the title. For some conditions, the concern was not of missing relevant papers but of using search terms that would yield false positives. Thus, the acronym SLI (used here for specific language impairment) is associated with 16 meanings in Wikipedia, as well as referring to genes in potatoes and worms. Attempts were made to restrict searches to exclude such usages, but it is nevertheless likely that the estimate for N publications is inflated by occasional errors. Another condition that was difficult to search was XXX trisomy, because XXX is used widely with a range of meanings. Conversely, the estimate for velocardiofacial syndrome will be slightly deflated because it was decided to exclude searches for papers with only the acronym VCF in the title, given that this acronym refers to a host of things other than velocardiofacial syndrome, and only a small minority of publications including it in the title were relevant. As noted above, identifying papers on intellectual disability was particularly problematic, and estimates of research on this topic are inflated because of the difficulty excluding articles on specific learning disabilities. It is noteworthy that nevertheless the amount of research on intellectual disability was below the level predicted from severity and prevalence (see Figure 1).

The second key variable in the analysis is prevalence. Estimates were taken from reputable published sources, but are bound to be imprecise for very rare disorders. Furthermore, for some disorders, especially those with multifactorial etiology, prevalence can vary substantially depending on the severity cutoff used. For instance, the prevalence of fetal alcohol syndrome disorder is estimated at 1 per 1000, but this condition is far less commonly identified in the UK than in North America, where a milder phenotype of ‘fetal alcohol spectrum disorder’ is recognised and estimated to be nine times as common [7].

A third key variable is severity, which was estimated according to the criteria from Table 2, averaging ratings across a range of experts. All raters agreed that this was difficult to do for those conditions with variable phenotype, such as tuberous sclerosis or autistic spectrum disorder. For a condition such as intellectual disability, severity will also depend on prevalence, with milder forms being more common than more severe. Because it was in general not possible to determine the severity of cases included in specific studies, average estimates were taken, but results for specific conditions need to take into account unreliability that is inevitable when using these fuzzy estimates. Furthermore, the severity scale was devised to assess how far the condition affected educational attainment and adult independence, but did not take into account physical symptoms that characterise some of the rarer conditions. Thus, the majority of females with Turner syndrome do not have significant intellectual impairment and live independent adult lives, and hence this condition is rated as mild. However, the syndrome involves cardiac problems and infertility, which are not trivial symptoms.

Despite these limitations, the analysis gave a striking replication of the findings on neurological conditions [1], namely that, when prevalence is taken into account, the number of publications on rare conditions is greatly in excess of that for common conditions. At the extremes of the distribution considered here, between 1985 and 2009 there were 428 publications on Lesch-Nyhan syndrome, which affects an estimated 57 children in the UK, compared with 340 publications on speech sound disorder, which affects over 1 million. However, these two extremes suggest a reason why this is so: more research is done on more severe conditions. Speech sound disorder can be marked and persistent, but it is usually a relatively mild condition that is often transient and may have little impact on a child's social or educational prospects, whereas Lesch-Nyhan syndrome has severe neurological and cognitive consequences, including self-mutilation as a striking symptom, and is associated with death from renal failure or hypotonia in childhood or young adulthood. Overall, it was possible to show that a high proportion of variance in number of publications could be accounted for in terms of severity.

Furthermore, the amount of research on a given condition will in part depend on the disciplines involved in investigating it. Given the large number of identified publications, it was not possible to classify the discipline of each one, but a rough index was obtained to estimate the proportion of publications that involved genetic investigations. Not surprisingly, these were more common for single-gene disorders, most of which were rare. The amount of genetic research on a disorder was also a significant independent predictor of publication index.

There were some intriguing exceptions where severity failed to predict research activity, which can be seen as outliers in Figure 1. Within the conditions with presumed multifactorial etiology, ADHD and autism spectrum disorder fell within the range of predicted research, with Tourette syndrome slightly above predicted levels. However, the publication indices for dyslexia, dyscalculia, developmental coordination disorder and speech sound disorder fell well below prediction. Dyslexia and SLI are comparable to ADHD in prevalence and severity, yet the number of publications for dyslexia was 4 times lower than for ADHD and that for SLI was 16 times lower than ADHD. It is also interesting to note that there were three times as many studies on developmental dyslexia as on SLI, and nearly ten times as many as on speech sound disorder, although these conditions are similar in terms of rated severity and prevalence. It is perhaps relevant that these latter two conditions posed particular problems when searching for publications, because of the variable terminology used to refer to cases, suggesting that diagnostic uncertainty might be a factor leading to low research rates. This is a field where there is considerable debate about how to diagnose and categorise cases, whether there are disorders that are distinct from normal variation, and where the same terminology that is used to refer to a disorder is also used to refer to symptoms in other disorders. Even though both disorders feature in the diagnostic classifications of ICD-10 [8] and DSM-IV [9], few researchers adopted definitions or nomenclature from the diagnostic manuals.

Another intriguing contrast is between Turner syndrome and Klinefelter syndrome, both caused by sex chromosome aneuploidies, and both affecting fertility. Table 3 indicates that there were nearly two times as many publications on Turner syndrome as on Klinefelter syndrome, even though Klinefelter syndrome is more than three times as common. Here the discrepancy is likely to relate to the ease of identification of affected individuals. The prevalence rates for Klinefelter syndrome are derived from prenatal and/or newborn surveys, but because the phenotype is not striking, many affected individuals are not identified in childhood. In contrast, the physical phenotype is far more obvious in Turner syndrome. This kind of explanation could also account for the dearth of studies on XYY and XXX trisomies; the majority of affected individuals are not identified because the phenotype is mild [10].

Even after taking into account severity and rates of genetic research, prevalence still was a significant predictor, with higher rates of publication for the rarer disorders. In considering reasons for the higher publication ratios in rare vs. common diseases, Al-Shahi et al.[1] noted that if the publication ratio for stroke were equal to that of variant Creutzfeldt­Jakob disease, clinicians and researchers interested in stroke would have had to read about 10 000 papers per week! One can turn this logic on its head and argue that if rare disorders had a publication index comparable to that for common disorders, there would be too few papers to give a critical mass of evidence in the area. For instance, if we extrapolate from the publication index for SLI (0.13 papers per 100 affected children over the 25 year period), for rare conditions where the national number of affected cases runs into hundreds rather than thousands, there would be no effective research literature. For the even rarer ‘orphan’ conditions with only a handful of affected individuals, lack of research is recognised as a serious problem [11]. The current analysis indicates that research activity on these disorders would have to occur at a rate well in excess of that predicted by extrapolation from more common disorders, in order to have a critical mass of publications and build research capacity.

The data reported here also suggest a positive effect of critical mass, insofar as the rate of growth in research on a disorder was related to the number of publications recorded in the first time interval, 1985–1989. This is compatible with the idea that a field develops by skilled researchers training their students and postdocs, who are likely to then study the same condition. If each mentor trains several others, then growth will be exponential rather than linear. Nevertheless, there were two conditions – ADHD and autism spectrum disorder – where the growth in research was unexpectedly high. For autism, there are two factors that could be implicated: first, broadening of the diagnostic criteria has led to a dramatic increase in diagnosis [12], and second, research funding has been directed to this area, for instance, by the Combating Autism Act passed in 2006 by the United States Congress, which authorized nearly 1 billion dollars over five years to combat autism and related disorders. For the more classic forms of autism, both prevalence and severity are comparable to Down syndrome, yet funding for autism is six times greater, and the slope showing increase of NIH funding over time is dramatically higher than for any other condition. It seems likely that government initiatives play a large role in explaining the extraordinary rise of publications in autism, though there are in addition several private foundations that provide substantial funding for autism research.

The other condition showing a very high increase in publications over time is ADHD, again with an associated high level of NIH funding. ADHD is comparable in prevalence and severity to SLI and dyslexia, and the proportion of genetic research on ADHD is relatively low, yet it attracts 19 to 21 times as much funding as these two conditions. Figure 2 suggests an explanation in terms of the professional disciplines with primary responsibility for these conditions. ADHD, like autism, is funded through several NIH institutes, but primarily by NIMH, suggesting most researchers in this field are psychiatrists. Dyslexia is funded mainly through NICHD and is the domain of psychologists, whereas SLI is funded mainly through NIDCD and is investigated principally by speech-language pathologists. This analysis suggests that inequities between professional disciplines in access to research training and funding may do a disservice to children who are affected by common yet under-researched neurodevelopmental disorders.

In summary, it is misleading to focus only on prevalence when comparing research activity for different conditions, as this suggests a massive excess of research on rare disorders. If severity is taken into account, the excess reduces considerably, although it is still present. This may, however, have to do with critical mass of research: there have to be proportionately more publications per affected individual for rare than common disorders, to ensure there is a body of work to build on. Nevertheless, even among conditions of similar frequency and severity there are some intriguing discrepancies in levels of research activity. It is suggested that these may have to do with extent of involvement of genetic researchers, specific initiatives in research funding, and lack of research funding in some disciplines working with neurodevelopmental disorders.
